# How paediatric nurses frame the ethics of non-disclosure directives

**DOI:** 10.1177/09697330251350383

**Published:** 2025-06-14

**Authors:** Mandy (Mervat) El Ali, Jenny O’Neill, Lynn Gillam

**Affiliations:** 6453 The Royal Children’s Hospital; 2281 The University of Melbourne; 95359 The Australian Catholic University, Melbourne; 6453 The Royal Children’s Hospital; 2281 The University of Melbourne

**Keywords:** Children, ethical dilemma, non-disclosure, paediatric nurse, truth-telling

## Abstract

**Background:**

Nurses caring for hospitalised children can be told not to disclose information to the patient. Such non-disclosure directives in adult care pose recognised ethical problems, as they impinge on a patient’s autonomy and right to their own information, and have been discussed widely in the literature, from a physician’s perspective. Despite the ethical implications, there is less discussion of the ethics of withholding information from children. Nurses are well positioned to advocate for the rights of a child while considering their best interests; hence, nurses’ thinking about the ethics of non-disclosure directives is valuable.

**Aim:**

The aim of this study was to explore the experiences and attitudes of nurses with truth-telling to seriously ill children, specifically how nurses frame and think about the ethical challenges when given a directive not to tell the truth to a child.

**Design:**

An interpretive phenomenological approach was employed for this research, with data collected by semi-structured interviews.

**Participant Population:**

Twenty-six nurses in Australia who had cared for children hospitalised with a serious illness in the previous 5 years.

**Ethical Considerations:**

Ethics approval was granted by the University of Melbourne’s Human Research Ethics Committee (37283A). Informed consent was acquired from all participants.

**Findings:**

Four themes encompass the views nurse-participants expressed about the ethics of a non-disclosure directive: (i) Lying is wrong, (ii) Children should know, (iii) It’s hard for us when the child doesn’t know, but (iv) It’s not our place to tell. Nurse-participants described how a non-disclosure directive affected how they cared for their patients.

**Conclusions:**

Nurse-participants believed they should be honest and articulated ethical reasons why children should be told the truth about their medical condition, but did not feel they were able to initiate this. It is recommended that nurses are supported in these ethically challenging situations and included in decision-making about how to respond when parents direct that information be withheld from their child.

## Introduction

Nurses sometimes encounter situations where they are told not to disclose specific information to a child who has been hospitalised for a serious illness. We use the term ‘non-disclosure directives’ to refer to these situations. Studies have explored ethical aspects of truth-telling to seriously ill children from the perspective of the physician, yet no published evidence represents an in-depth investigation of nurses’ ethical thinking when confronted with a non-disclosure directive. This paper will present findings from an Australian study that explored the experiences and attitudes of nurses caring for seriously ill children when given a non-disclosure directive, focusing on nurses’ ethical thinking.

## Background

A non-disclosure directive presents nurses with an ethical dilemma where nurses’ values may be challenged. In a paediatric setting, a directive to withhold poor diagnosis and prognosis from a child is not uncommon.^1^ Even in an adult clinical setting, where information is more readily disclosed, some family members of adult patients will also request health care professionals withhold a poor diagnosis or prognosis.^2–4^ The ethical requirement for clinicians to tell adult patients the truth about their diagnosis, prognosis and expected treatments^5^ is now widely accepted, as part of respect for patient autonomy and the need for informed consent.^6^ This is in alignment with national and international codes of ethical practice and supports participation in health care.^6^ Telling the truth to children is a somewhat different matter. The idea that children, especially younger children, should be told the truth has grown slowly over time, is becoming increasingly accepted, but is not fully established as a standard ethical position.^7^ This is because informed consent is not directly relevant to children, except for those who are Gillick competent (mature minors).^8^ For all other children, consent is given by their legal decision-maker, usually the parent/s. There are however ethical reasons beyond informed consent for telling the truth to children.

Hudson et al. (2019)^9^ explore some of the ethical reasons to disclose information to hospitalised children who are below the age where they would be considered mature minors. These include: respect and promotion of the child’s involvement in decision-making, promotion of involvement in the therapeutic relationship, avoiding restriction of future autonomy, respect for the child as a person, improvement of the well-being of the child and to epitomise truthfulness and fidelity.^9^ Gupta et al. (2008) argue that truth-telling to patients is a moral obligation and even in very young children is important to develop their emerging autonomy.^10^ Others argue that honesty is an essential ingredient in the development of trust,^7^ and the establishment of a therapeutic relationship^1^ with children who are seriously ill, which then ethically obliges nurses to tell children the truth.^5,11–13^

However, truth-telling to children is not universally supported or practised. Some studies have shown physicians agreeing to a non-disclosure directive initiated by a child’s parent, even completely falsifying information given to children, in support of the parent’s belief that it would be better if the child didn’t know.^10,14^ Nurses also engage in behaviours where information is omitted or manipulated^15^ during direct patient care, referred to as benevolent deception.^16,17^ This practice is illustrated in a study by Tuckett (1998) who describes a situation where a nurse admitted to using deception to ensure a child took their medication. This action was justified by the nurse, who stated she had parental permission, and emphasised the importance of children receiving their prescribed treatment by any means.^15^ In some cultures and contexts, lying or deception is believed to serve a purpose in relation to beneficence and non-maleficence,^18^ because the truth would cause fear and anxiety, and extinguish hope of cure and recovery, thus causing harm.^15^

Overall, the ethics of non-disclosure to children is not clear-cut. Ethical reasons can be given in favour of withholding information, especially for children who lack full autonomy. Sigman et al., (1993), recognises this but argues that it would need a very strong reason to justify not telling the truth to a patient.^19^ Gillam et al (2022), writing specifically about children, frame the ethical issue about truth-telling in terms of the child’s interests, leaving open the possibility that truth-telling can have both positive and negative effects on a child’s interests.^7^

Nursing codes of ethics do not provide explicit guidance about telling the truth to children.^20^ The International Nursing (ICN) Code of Ethics and Code of Conduct states that nurses must ‘ensure that the individual and family receive understandable, accurate, sufficient and timely information in a manner appropriate to the patient’s culture, linguistic, cognitive and physical needs, and psychological state on which to base consent for care and related treatment’.^20^ This is very general, links information giving with consent, and does not address directly the issue of telling the truth to children. Other nursing codes, such as the Canadian^21^ and UK^22^ codes, explicitly emphasise truth-telling and honesty. Hence, when faced with a situation where they are given explicit instructions to not disclose medical information to patients, nurses are likely to experience moral conflict.^23^ However, these documents do not directly address paediatric patients. They could be interpreted to apply to patients of all ages, but it could also be argued that the ethical reasons for truth-telling to children are inherently different to those for truth-telling to adults, and thus the guidance to nurses is not clear.^3,4,6,24^

## Aims

The aim of this study was to explore the experiences and attitudes of nurses with truth-telling to seriously ill children, specifically how nurses frame and think about the ethical challenges when given a directive not to tell the truth to a child.

## Methods

### Research design

This was an interpretive phenomenological study,^21,25,26^ exploring the lived experience of nurses looking after seriously ill children. The aim of this study was to explore the experiences and attitudes of nurses with truth-telling to seriously ill children, specifically how nurses frame and think about the ethical challenges when given a directive not to tell the truth to a child. The study sought a rich interpretation of the lived experience of the nurses from the accounts they gave.

### Research participants

Purposive sampling was achieved through the implementation of a Qualtrics survey to ensure the participants met the following inclusion criteria:

(1) Australian registered nurses at least 12 months post-graduation
(2) Had cared for children between the ages of 4 and 12 hospitalised with a serious illness within the preceding 5 years.

Recruitment occurred between November 2019 and October 2020 by email, via a national professional body for paediatric nurses, and then through snowballing and social media platforms.

### Sample size

Recruitment for this interpretative phenomenological inquiry was guided by meaning saturation which is explained by Hennink et al. (2017) as ‘the point when we fully understand issues, and when no further dimensions, nuances, or insights of issues can be found’^26^ (p. 594). Both code and meaning saturation aim to support the rigour and understanding of the qualitative inquiry, but while code saturation only requires a small number of interviews to identify patterns and concepts in the data set, meaning saturation requires a larger data set (16–24) to provide understanding of the identified concepts.^22^

### Data collection

Data were collected through semi-structured individual interviews, using an interview guide (see Table 1) based on key ideas and themes presented in the literature. A pilot interview was conducted with a senior nurse to check appropriateness of questions and prompts in the interview guide. All interviews were conducted by the first author and principal researcher who is a paediatric nurse in an academic setting.

**Table 1.** Examples of interview questions.

Main question	Prompts
General views	
What is your general view about the sort of information that children get about what will happen to them in hospital?	Can you think of situations where you have thought that a child is being given too much or too little information about their medical condition, procedures, treatment, risks of treatment?
Answering a child’s question	
Have you experienced a situation where a child asked you a question (which you knew the answer to), and you felt uncomfortable/ uncertain or questioned yourself on how to respond?	What made you think that the information was too much or too little?
	What made you feel uncomfortable about the situation? What was troubling to you?
Information being withheld from a child	
Have you experienced situations where information is being deliberately withheld from a child?	Could you describe the situation?
	Why do think this happened?
	How did you feel about the situation?

Twenty-seven nurses were interviewed, two face to face and 25 online due to the lock down of the COVID-19 pandemic in 2020. The interviews were all audio-recorded and transcribed verbatim. One participant withdrew before data analysis leaving 26 semi-structured interviews for analysis.

### Data analysis

Data were analysed using Braun and Clark’s reflexive thematic analysis which includes becoming familiar with the data, generating initial codes, searching for and reviewing themes, and defining and naming themes to support an emergent phenomenon.^27^ In this study, analysis was supported through the use of Dedoose,^28^ a qualitative analysis platform that allows, tracking, auditing of data and collaboration of multiple researchers.^28^ To ensure that the analysis represented an authentic interpretation of the data, the transcripts were read, and re-read, and a subset co-coded by all authors, allowing patterns and concepts to be identified robustly, guiding the consolidation and presentation of themes that emerged from the data. According to Smith et al. (2022) this method of phenomenological analysis is committed to examining how ‘people make sense of major life experiences’^29^ (p. 1). Smith et al. (2022) state that interpretative phenomenological analysis engages a double hermeneutic ideology as the researcher seeks to interpret the participants’ interpretation of their lived experience.^29^ The researchers engaged in this process as a method of seeking to understand the participants’ lived experience.

### Rigour

Trustworthiness in naturalistic inquiry is essential in presenting what the data represents. According to Lincoln and Guba, rigour in qualitative research is achieved by addressing truth value, applicability, consistency and neutrality, ensuring robust and credible findings.^30,31^ In this study, this was achieved through meticulous data tracking and audit trails through electronic note keeping, diary entries and briefing between the researcher and the supervision team, and co-coding. The researcher and supervision team undertook regular briefing during the data collection process to ensure biases were identified and noted and the researcher ensured awareness of any potential biases prior to each interview ensuring credibility. Transparency in the research methodology including the purposive sampling, recruitment note keeping, tracking and the collaboration in coding through the data analysis process ensured transferability.

### Ethical considerations

The research study received ethics approval by The University of Melbourne’s Human Research Ethics’ Committee HREC (37283A). The participants in the study were provided with a plain language statement outlining the research protocol that included potential risks of participating in the study. Despite the study being classified as low risk, the researcher prepared a Study Distress Protocol and safety plan, and the voluntary nature of the participation was reiterated at the commencement of each interview.

## Results

Twenty-six semi-structured interviews of between 28 and 68 minutes were conducted, providing a rich source of data. The participants worked in a variety of clinical settings caring for children. Their years of experience as well as their expertise were rich in diversity, as shown by demographics provided in Table 2.

**Table 2.** Participant demographics.

Participant Code	Nursing role at time of interview	RN experience in years	Postgrad studies	Employment area	Age range
P1	Advanced practice nurse	16+	Yes	Other	30–39
P2	Advanced practice nurse	16+	Yes	Complex care	30–39
P3	Bedside nurse	1–5	No	General ward	21–29
P4	Advanced practice nurse	16+	Yes	Primary care	30–39
P5	Bedside nurse	6–10	No	Other	30–39
P6	Bedside nurse	16+	Yes	PICU	40–49
P7	Advanced practice nurse	6–10	No	Complex care	21–29
P8	Bedside nurse	6–10	Yes	PICU	21–29
P9	Bedside nurse	6–10	No	General ward	30–39
P10	Nurse clinical education^a^	11–15	Yes	Other	30–39
P11	Bedside nurse	16+	No	General ward	60–69
P12	Primary care nursing	11–15	Yes	Other	30–39
P13	Primary care nursing	1–5	No	General ward	21–29
P14	Primary care nursing	6–10	No	Primary care	21–29
P16	Nurse clinical education	16+	Yes	Other	30–39
P17	Primary care nursing	6–10	No	Primary care	30–39
P18	Bedside nurse	1–5	No	General ward	21–29
P19	Bedside nurse	16+	Yes	PICU	30–39
P20	Bedside nurse	6–10	No	General ward	30–39
P21	Bedside nurse	6–10	No	PICU	30–39
P22	Nurse clinical education	16+	Yes	Complex care	50–59
P23	Advanced practice nurse	11–15	Yes	PICU	30–39
P24	Bedside nurse	6–10	No	General ward	30–39
P25	Advanced practice nurse	11–15	No	Complex care	30–39
P26	Bedside nurse	16+	Yes	Other	60–69
P27	Bedside nurse	6–10	Yes	Other	30–39

^a^Includes undergraduate and postgraduate nurse educators and clinical educators working across hospital wards and departments.

**Figure 1.**
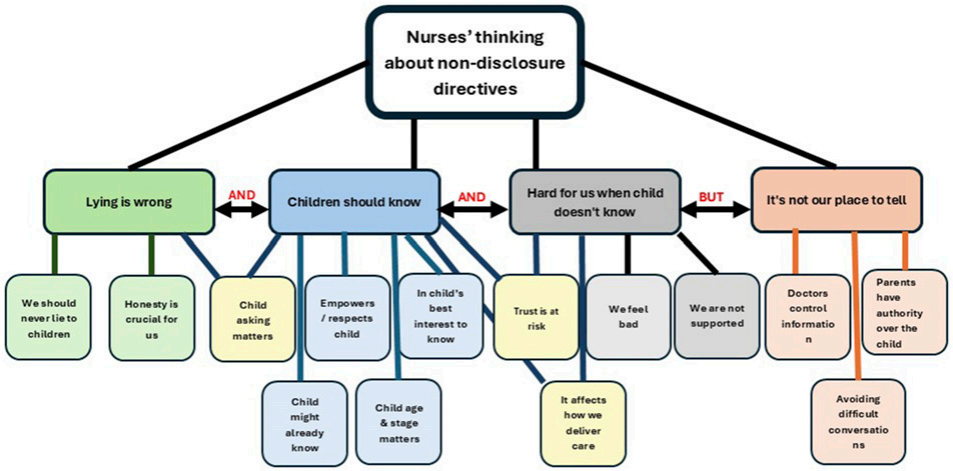
The phenomenon.

Nurse-participants described situations in which they had personally experienced being directed not to tell the truth to a child-patient and spoke at length about their thoughts and feelings about these situations. These personal accounts have been reported and analysed in a previous paper.^32^ The findings presented in this paper specifically relate to the ways in which the nurses framed and described the ethical aspects of non-disclosure situations. Participants’ ethical thinking about non-disclosure directives relating to children encompassed four main themes, built up from 14 sub-themes (see Figure 1), providing a complex picture of the way nurses frame the ethics of truth-telling to children. Nurse-participants strongly expressed the view that lying is wrong, and that children should know. They also emphasised that it is difficult for the nurses when the child doesn’t know. However, they believed that it was not for them to tell children the information that was being withheld from them.

### Lying is wrong

#### We should never lie to children

Discussion of lying specifically, as distinct from non-disclosure, was common across the interviews. Lying was regarded very negatively.

I have never seen any benefit ever, to lying to a child, I think we do a detrimental impact to these kids. (P23)

Nurse-participants explained the importance of never lying to a child by indicating the potential impact the deception and withholding directive had on the clinician-child relationship. As paediatric nurses, they saw that effective care relies on therapeutic communication and rapport with the child and family.

You don’t lie to kids, because… children, remember, they know, and then you lose trust in kids, and it just doesn't set the scene for a good… therapeutic relationship with a child. (P16)

The centrality of the child in the nurse-participant’s considerations was evident, with one nurse describing lying as meeting the needs of the parents and medical team with no benefit to the child:

That’s what I think is the worst part is how we often lie blatantly lie to kids about things that we know aren’t true, and I don't really know why we do it, because I mean… I know why we do it, but it doesn’t fulfill any of the child's needs, I feel like it just fulfils the medical and the parent’s needs. (P18)

#### Honesty is crucial for us

Nurse-participants presented honesty as part of their personal and professional value system. They saw honesty as a foundational value of morality, as indicated in this quote:

It just feels a bit immoral …For the people that are supposed to be… looking after you and the people that love you, ...to not be honest with you. I just feel it’s not very nice. (P9)

They also connected honesty with children’s understanding of what is happening to them:

We need to be honest with our children with what we’re doing, and give them the opportunity to comprehend what’s going on. (P23)

Some participants expressed the value of honesty in personal terms, such as ‘I’m very honest and open’ (P3) and ‘in my own family life… honesty is the best policy’ (P6). Others connected honesty with their professional knowledge and role:

But that was really challenging and difficult as an RN, caring for this child, like you want to be as open and honest as you can, and as truthful as you can. (P10)

and

I would like to be able to just be honest with these kids based on what I know, like where my knowledge of her health is at and never have to feel like I have to lie to her. (P18)

#### When the child is asking, it really matters

The ethical importance of honesty was seen to be emphasised in situations where children asked questions.

She was asking… will I ever walk again, and I didn't actually ask the parents’ permission at that time because I felt well, you want to be honest, in the context that they can understand, and I said No, sweetie, your legs, … and body is broken down there. (P6)

Nurse-participants expressed how children sometimes waited for their parents to leave the clinical setting to ask the tough questions. Even non-verbal children using communication boards asked the hard questions to the nurse, saying that they thought their parents wouldn’t tell them. Nurse-participants agreed that not responding to children’s direct questions can be harmful as it creates fear of the unknown.

*…* kids are so good at waiting for parents to go away to ask the true questions that are bothering them. And I don’t think it’s my responsibility.... but if I have a child who is asking questions, I think me ignoring those questions or deflecting those questions is just as harmful. Because you've got a child who is openly seeking some psychological support network… (P23)

Nurse-participants also reported that as children got older, they asked more questions, particularly those who had serious illness.

And that was where I first started encountering that, I guess that older age group where yes, children who either had cancers or trauma or a cardiac condition that was palliative, where they’re starting to ask more questions. (P6)

### Children should know

The theme *Children should know* encapsulates nurses’ statements about the reasons children should be told the truth. There was consensus about the importance of the child understanding what is happening to them to avoid confusion, fear and other harms or detriments.

I’ve been in lots of procedural type situations where children are so anxious about what’s coming next. I think sometimes if we were better at setting them up for it… when it gets to the point that you’re holding children down, and some children are just scared… but perhaps if we were better… making sure they knew why we were doing something. (P1)

Nurse-participants expressed that information was empowering to the child as well as affording respect to the child. They also believed that some level of consent was necessary as part of respect for the bodily autonomy and privacy for children.

… we are violating their body in some way with everything that we do…. if we’re not asking for permission to put a sats probe on a child’s finger, why should they ever feel like they have the authority to say no to that circumstance? (P23)

#### In child’s best interest to know

Nurse-participants expressed a general consensus that it is in the child’s best interest to be told the truth. In a practical context, nurse-participants stated that any invasive procedures children need to endure as part of treatment should be justified to them, and this requires giving information about what is happening to them.

I think when people have answers about why they’re experiencing something, and why … that child’s experiencing pain, to understand why that’s gonna relieve her, I guess, give her some sense of calm, or know why it’s happening, because they can comprehend information. (P12)

In addition, not telling the truth causes harm by setting children up to potentially distrust health care professionals. When children do not know,

… it…. Generate(s) a child who then is very fearful of coming to the doctor because the last time they came was you know; they didn't know what was going to happen… (P5)

Some nurse-participants expressed the belief that when children don’t have information, they can create an explanation in their own mind that is worse than the truth.

But again, I think you know, when they say nothing, kids go, well, then it mustn’t be nothing because you’re crying … and kids just figure stuff out and from what’s going on around them to the point where they either figure out what’s going on, or they… think something far worse than what is actually happening. But sometimes the truth is actually better than what they can imagine... (P4)

#### Child might already know

Some nurses suspected that the child may already have some knowledge of their medical status, and this added to the impetus to tell. One stated that terminally ill children potentially know they are very ill and dying.

… they gave her little bits of information, and they never told her that she was going to die. But she was sort of…quite smart, three-year-old and was slowly figuring it out anyway. (P6)

Nurses expressed that where the child gives an indication that they may know, they assumed that the child was protecting their parents from knowing that they know. One nurse-participant relayed a dream described to her by a terminally ill child who knew she was dying.

I've been dreaming about Grandma and Grandpa, and they tell me they’re waiting for me. And that it won’t be very long. And we'll be all together in heaven.…but I think it’s going to take a long time... because Mommy and Daddy won't let me go. (P11)

#### The child’s age and stage matters

Most nurse-participants discussed the importance of information being age-appropriate and being cognisant of diversity in children’s background and understanding when making a decision of what and how to tell.

They’re at an age where I think that perhaps… they should be part of it, whether or not they’re… going to be able to fully make all of the decisions themselves… Most 12-year-olds, especially ones that have got a diagnosis of some description, or… some kind of complex illness… usually do have their own sort of wishes, and they do have a level of autonomy around … what they want their life to look like. (P7)

and

Look, I think… five, six can be tricky because … I still think you eventually have to be told, but it certainly might be better for a parent to tell them… Whilst I can sit here and give you an age, this is dependent on so many different factors, you know. (P16)

Nurses-participants indicated that as soon as the child was cognitively aware, they should be afforded the right to their own medical information, at least to some extent. However, nurse-participants were more emphatic about telling the truth to teenagers than to younger children, consistent with their maturity and ability to make more sophisticated decisions:

But at the same time, yes, when you got a young adult, who, I guess I felt had the right to know that her life is, is short. It’s, it’s sad to cheat them of that knowledge. (P6)

### Hard for us when the child doesn’t know

Nurses felt that trust would be significantly affected when there were constraints on what they could and couldn’t say in front of the child. Ultimately, nurse-participants described how a non-disclosure directive affected the way they delivered care.

#### Trust is at risk

When asked to withhold information, nurse-participants described the effect it had on the trust in the therapeutic relationship.

Oh, it’s awful because you feel like you're the nurse and you want them to have trust and faith in you. And you know, you build your whole… career … around building trust with the kids, because they’re so… frightened… and then you know you’re maybe putting a strain on that trust relationship. (P17)

Another nurse-participant expressed:

…challenges with things like that is you’re being asked to not divulge information, and not that you would necessarily say to a 10- or 11-year-old, specifics, but … then being asked to be really careful with what you say… I do think that impacts rapport building. (P16)

#### It affects how we deliver care

Nurse-participants described strategies they would implement to manage the non-disclosure directive, which resulted in them providing less than optimal care to the child. Avoidance was a common strategy – avoiding the child, or avoiding the topic, both of which had negative implications for the child.

… so, you close yourself off … maybe you go into the room less often, or you because you don’t want to lie not even lie but you don’t you feel like… withholding something from them, and so you don't want to spend too much time or build that rapport or relationship with them in case, those hard questions are asked. (P20)

and

All the conversations became… do you need to go to the toilet, …how are you feeling at the moment? Was all just very focused on what needed to be done? (P7)

It also made routine nursing tasks like giving medication feel fraught:

So that made treating him really hard because if mum had left, and we were trying to give medications, and you were trying to talk to him about what we were doing, you have to do it in a kind of not sneaky, but in a way where it was like you would tell him what you were doing, but not 100% disclosed, because he was the child; mom was his carer and we had to kind of respect her wishes. (P10)

#### We feel bad

When nurse-participants were asked how they felt when they were told to withhold medical information, they consistently described feeling bad.

‘I don’t know if you could put a word on the feeling. It’s like a wrestling within you… like a feeling of unsettledness where you… feel like it’s not your place to make the final decision. You… feel dishonest when a child is searching for that information. (P6)

Nurse-participants described the tension that builds in the room when they have been told not reveal information to a child:

… there’s sort of like, it's almost like the rooms holding their breath… where… there’s just no way to relax, and I’m not saying that if your child were dying, that you would be relaxed, but it’s just a different type of tension. (P20)

Worry about accidentally revealing the forbidden information was described by several nurse-participants:

I don’t know that I can be that conscious of what I’m saying… I’d be petrified of letting it slip, but…also… kind of relieved if I did let it slip because then she knows. Obviously then there’s the repercussions from the family and gosh, that is a horrible situation. (P25)

#### We are not supported

Nurse-participants also expressed the lack of support they received when put in a situation where they were expected to care for a child and family throughout a shift with limitations on what they were allowed to say in front of the child and how they were expected to respond to questions.

… it’s not something that just goes away, and we don’t debrief, we don’t talk about it… we’re just expected to get on with it and do what the plan says and know how to react in those situations. We just are expected to know how to not say anything or not have those conversations and … it’s exhausting. (P18)

### It’s not our place to tell

Despite their ethical ideas about the importance of honesty and being truthful with children, and the difficulty of managing the care of a child within a withholding scenario, nurse-participants did not believe that they should give information to a child regarding their child’s diagnosis or prognosis in a situation where a non-disclosure directive had been made. There were several reasons for this.

#### Parents have authority over the child

Nurse-participant stated that the transfer of information is guided by what the parents want.

Yeah, so a lot of what we do, we’re guided by the parents, and this is what we find a bit tough. Some parents are very, against us saying anything at all. (P2)

They took the view that parents’ decisions needed to be respected.

I think, as a nurse, you have to respect those wishes, because they are her legal guardian. (P10)

I think morally ethically… she’s still a minor, … she’s still under the care of the parents if parents choose not to let her know, we have to respect that as the nurse. (P26)

During the interviews, nurse-participants were asked if they believed certain cultures were more likely to implement a non-disclosure directive, and while there was no consensus from the nurse-participants regarding this, one participant stated that sometimes health care professionals used a family’s culture as an excuse to withhold information.

I think we often played into that cultural aspect without really challenging the family… and they never wanted him to know… we got to a place where his condition was palliative, and that there was nothing else we could do for him. And even on his final moments, they refused to let him know that we were palliating him and that we were increasing sedation and keeping him comfortable. (P23)

The nurses implied that had the family been from an English-speaking, highly educated background, there may have been more effort to persuade parents to have an open conversation with the child.

#### Doctors control information

There was a sense from the nurses that doctors own the medical information, and that only they have a legitimate stake on disclosing diagnosis and prognosis for the first time. Nurse-participants were however happy to have an open conversation after the doctor had attended and had the initial conversation.

I don’t think it’s my place to talk about the results, … but… I wouldn’t be uncomfortable about discussing it with them, particularly after the doctors have been out of the room, if they’ve sort of said, ‘Yep, these are the results’, and then go away, like, totally happy to have the conversation… a diagnosis talks, I think is out of my scope as a nurse. (P20)

#### Avoiding difficult conversations

As a result of these beliefs about nurses not having legitimate authority to give information, nurse-participants avoided situations were that information might come up.

I just remember him saying ‘I'm sick’, and that was kind of it. But I never explored it any further, because I didn’t want it to go down the path. (P10)

When the conversation became very difficult, it was avoided all together: ‘There was very little talk about death’ (P22).

## Discussion

The findings demonstrate how nurse-participants frame the ethical challenges they experience when given a non-disclosure directive. While ethical conduct is embedded in the registered nurses’ standard for practice,^33^ nursing codes of ethics do not provide explicit guidance about telling the truth to children.^20^ Nevertheless, nurse-participants in this study did express ethical views on non-disclosure directives, in terms clearly related to general ethical principles, as envisaged by the American Nurses Association Code of Ethics, which states that ‘Beyond professional codes of ethics, standardly accepted principles of health care ethics support the ethical importance of telling the truth to patients’.^34^ Nurse-participants gave a number of reasons why children should be told the truth, which align with the ethical considerations put forward in the work of Hudson et al. (2019).^9^ For example, Hudson et al. (2019) refer to the ethical importance of involving the child in the therapeutic relationship, a consideration which many nurse-participants in our study highlighted. Our participants also pointed to the way that children are more calm and less fearful when they understand what is happening to them (see the sub-theme ‘in child’s best interests to know’), the same ethical point as Hudson et al. (2019) ‘promoting the well-being of the child’.^9^ Gillam et al. (2022) identify specific interests of children that are typically advanced by having access to information, including sense of control, sense of self, and relationship with parents.^7^ Some of our participants did talk about best interests and referred to children having knowledge and understanding of their medical situation and treatment as important for children’s best interests. They also pointed to involvement in medical decision-making, especially for the older child and adolescent who are already making other sorts of decisions about their lives, as an ethical reason not to withhold information. In the ethics literature, this is termed ‘respect for developing autonomy of the child’.^35^ Children’s right to know and right to participate in decisions, another prominent idea in these current ethical literature, was specifically raised by some nurse-participants.^36,37,38^ For children with more developed cognitive capacity to understand the language and ideas required to participate, the nurse-participants expressed more ethical concern in having to withhold information from them. A child’s rights are not determined on the basis of their age,^38^ and the competence of a child to participate in their care is dependent on their cognitive abilities.

So, the ethical thinking of nurses in our study was congruent with many aspects of the current ethical literature on truth-telling to children, although they mostly used more everyday language, rather than technical ethics terms, in expressing these ideas.

## The centrality of honesty and not lying

The most notable feature of nurse-participants’ ethical thinking in this study was the considerable ethical value they placed on honesty. Although the concept of honesty as a moral attribute is not new,^39^ and others have articulated truthfulness and veracity as among the ethical values supporting truth-telling to children,^12^ our study highlighted how much weight nurses put on honesty. This implies honesty is a fundamental principle that guides nurses’ practice and maintains their integrity. This matches the prominence of honesty and related moral virtues in nursing codes of ethics. The Canadian Nurses Association code states that ‘nurses are honest and practise with integrity in all of their professional interactions’.^21^ In the United Kingdom, the professional standards of practice explicitly link honesty with professionalism by presenting the concept of veracity under the promotion of professionalism and trust.^22^ A literature review published in 2013 identified 10 common ‘nursing ethical values’.^39^ Honesty was included under the values of trust and human relationships, which make up the ingredients of the therapeutic relationship. The authors emphasised that these values reflected the foundation of the nursing profession.

One problem with using the term ‘honesty’ to talk about the ethics of non-disclosure directives is that it does not clearly distinguish between lying (saying false things) and not saying what is true (sometimes called ‘lying by omission’).^40^ In the philosophical literature, a clear account of whether honesty allows or forbids omitting the truth is not readily found. Across many philosophical texts, honesty is referenced as a virtue^41–43^ and described as the moral action of truth-telling, but the status of lying by omission is not addressed.

In Tuckett’s 1998 study of nurses’ practices around lying and deception,^15^ nurse-participants admitted to half-truths and denials as deceptions rather than lies. The author concluded that ‘lying is understood only as a misleading statement and never an omission, silence or evasion that otherwise may mislead’.^15^ He states that ‘in the nurse’s view, only by committing herself to speech does she lie’. Nurses in Tuckett’s study did sometimes choose to lie, based on their understandings of the nurse–patient relationship and notion of caring.^15^ They made an individual assessment to lie for reasons of beneficence and non-maleficence but acknowledged the negative outcomes of maintaining the lie long-term denying the patient the ability to ‘face the reality of the situation’^15^ (pp. 297).

Tuckett’s study did not focus on any specialty clinical setting, and the majority of the situations discussed by participants were in the context of their care and therapeutic relationship with adult patients. In our study, nurse-participants did not describe any instances of deciding to lie. It was clear that lying (actively saying something that is not true) was particularly problematic for their perception of honesty. They felt morally obliged to be honest and so avoided encounters where they may be forced to lie. It is interesting to consider whether the paediatric context of our study is related to this difference from Tuckett’s findings. It is possible that nurses who care for children feel more obliged to be honest than nurses working in an adult setting. The difference between truth-telling in adults and truth-telling in children could be the subject of further research.

## The role of hierarchy and power

Nurse-participants in our study agreed that it was not in their scope to disclose medical information to a child where parents implemented a non-disclosure directive. This is similar to a study published in the 90s, where nurses expressed that disclosure was not within the ‘nurses’ remit’^44^ (pp. 1364) but expressed a preference for honesty and open disclosure to terminally ill patients. In our study, nurse-participants believed that although they were ethically bound to be honest, institutional constraints in the form of their assumed scope of practice (i.e. the belief that doctors own the medical information) excused them from upholding the virtue of honesty. The hierarchical and systemic structure of health care, and the physician’s significant power in terms of decision-making and approval of plans of care, is widely recognised.^44,45^ According to Ricciardelli et al. (2022), this power can extend to the suppression of decision-making capabilities of nurses.^45^ It was evident in our study that nurses were not part of the decision to withhold medical information from children, even though they would be the ones most directly involved in the process of withholding the information. It was also notable that nurse-participants did not report challenging the decision made by doctors to support parent-initiated non-disclosure directives. This can be explained by Lupton’s idea that the institutional power dynamic is maintained not just by doctors but also through nurse self-regulation, with nurses adhering to the expectations within the medical hierarchy and deciding not to challenge.^46^

Hierarchy and perceived scope of practice appeared to be the strongest factor in determining what nurses did when a non-disclosure directive was given. Although some nurse-participants discussed as an ethical reason for upholding non-disclosure directives that parents’ decisions about their own children carry ethical authority and should be respected, this seemed to be less influential overall than the hierarchy and scope of practice issues, which are not fundamentally ethical considerations.

## Conclusion

Our study was limited to paediatric nurses working in Australia. Nevertheless, it did cover a variety of practice settings and included nurses with different levels of experiences. It showed some clear and consistent themes which may well be relevant to paediatric nursing practice in other countries, especially those with a relatively similar health care system and population mix.

Our study showed that when a non-disclosure directive is given in a paediatric setting, nurses may experience a conflict, challenging their morals and values. Specifically, nurses are likely to be ethically opposed to lying to children, based on strongly held values in favour of honesty. Nurses in our study identified several reasons why children should be given information, which they related to doing what was in the child’s best interests. This meant that they felt that children were being let down or not being cared for in the way they deserved to be when information was being withheld from them. This would obviously create a difficult environment for nurses to work in, and yet they indicated that they received no support in situations where they were expected to uphold a non-disclosure directive. They generally accepted that it was not their place to be the ones to disclose information that the child did not know, but this did not mean that they found upholding a non-disclosure directive easy. Our findings vividly show the many difficulties they encountered, and the impacts on the way they could provide care to children.

### Recommendations

The main recommendations we make are (1) that nurses be provided with support in situations where they are directed not to disclose certain information and (2) that nurses be involved along with doctors in making decisions about how to respond when parents direct that information be withheld from their child. Parents’ directives should not simply be accepted without discussion or regarded as unchangeable. Clinicians should support parents and families in the clinical setting in facilitating developmentally appropriate truth-telling practices to seriously ill children, and nurses should be an integral part of this process.

## Data Availability

The participants of this study did not give written consent for their data to be shared publicly, so due to the sensitive nature of the research, supporting data is not available.

## References

[1] El Ali M Licqurish S O’Neill J Gillam L . Truth-telling to the seriously ill child - Nurses’ experiences, attitudes, and beliefs. Nursing ethics; 2023: 9697330231215952–9697330231215952. DOI: 10.1177/09697330231215952. PMC11370149 38128903

[2] Ben Natan M Shahar I Garfinkel D . Disclosing bad news to patients with life-threatening illness: differences in attitude between physicians and nurses in Israel. Int J Palliat Nurs 2009; 15: 276–281. DOI: 10.12968/ijpn.2009.15.6.42984. 19568214

[3] Bou Khalil R . Attitudes, beliefs and perceptions regarding truth disclosure of cancer-related information in the Middle East: a review. Palliat Support Care 2013; 11: 69–78. DOI: 10.1017/S1478951512000107. 23171758

[4] O’Sullivan E . Withholding truth from patients. Nurs Stand 2009; 23: 35–40. DOI: 10.7748/ns.23.48.35.s49. 19753871

[5] Tuckett AG . Truth-telling in clinical practice and the arguments for and against: a review of the literature. Nurs Ethics 2004; 11: 500–513. DOI: 10.1191/0969733004ne728oa. 15362359

[6] Beauchamp TL Childress JF . Principles of biomedical ethics. 8th ed. New York, NY: Oxford University Press, 2019.

[7] Gillam L Spriggs M McCarthy M Delany C . Telling the truth to seriously ill children: Considering children’s interests when parents veto telling the truth. Bioethics. 2022. 10.1111/bioe.13048 PMC9542183 35590446

[8] Griffith R . What is Gillick competence? Hum Vaccines Immunother 2016; 12: 244–247. DOI: 10.1080/21645515.2015.1091548. PMC4962726 26619366

[9] Hudson N Spriggs M Gillam L . Telling the truth to young children: ethical reasons for information disclosure in paediatrics. J Paediatr Child Health 2019; 55: 13–17. DOI: 10.1111/jpc.14209. 30198118

[10] Gupta VB Willert J Pian M , et al. When disclosing a serious diagnosis to a minor conflicts with family values. J Dev Behav Pediatr 2008; 29: 231. DOI: 10.1097/DBP.0b013e31817996ab. 18550993

[11] Higgs R Ingram L Atkin M , et al. A father says ‘don’t tell my son the truth’ [with commentary]. J Med Ethics 1985; 11: 153–158. 4057221 10.1136/jme.11.3.153 PMC1375181

[12] Muñoz Sastre MT Sorum PC Mullet E . Lay people’s and health professionals’ views about breaking bad news to children. Child Care Health Dev 2014; 40: 106–114. DOI: 10.1111/j.1365-2214.2012.01420.x. 22928950

[13] Xue D Wheeler JL Abernethy AP . Cultural differences in truth-telling to cancer patients: Chinese and American approaches to the disclosure of 'bad news. Prog Palliat Care 2011; 19: 125–131. DOI: 10.1179/1743291X11Y.0000000004.

[14] Antoun J Saab BR . A culturally sensitive audiovisual package to teach breaking bad news in a Lebanese setting. Med Teach 2010; 32: 868–869. DOI: 10.3109/0142159X.2010.524520. 20879162

[15] Tuckett AG . Bending the truth: professionals narratives about lying and deception in nursing practice. Int J Nurs Stud 1998; 35: 292–302. DOI: 10.1016/S0020-7489(98)00043-1. 9839188

[16] Pourgholam N Shoghi M Borimnejad L . Patients’ lived experiences of the paternalistic care behavior: a qualitative study. J Caring Sci 2022; 11: 163–171. DOI: 10.34172/jcs.2022.10. 36247035 PMC9526793

[17] Zolkefli Y . The ethics of truth-telling in health-care settings. Malays J Med Sci 2018; 25: 135–139. DOI: 10.21315/mjms2018.25.3.14. 30899195 PMC6422557

[18] Kopelman LM . Multiculturalism and truthfulness: negotiating differences by finding similarities. S Afr J Philos 2000; 19: 51–64. DOI: 10.1080/02580136.2000.10878202.

[19] Sigman Garry S Kraut Jerome La Puma John . Disclosure of a diagnosis to children and adolescents when parents object: A clinical ethics analysis. Am J Dis Child 1993; 147(7): 764–768. 10.1001/archpedi.1993.02160310066020 8322748

[20] International Council of Nurses [ICN] . The ICN code of ethics for nurses: Revised 2021. Geneva, Switzerland, 2021.

[21] Schneider ZM . Nursing research: methods, critical appraisal and utilisation. 2nd ed. Sydney: Mosby, 2003.

[22] Hennink M Kaiser BN . Sample sizes for saturation in qualitative research: a systematic review of empirical tests. Soc Sci Med 2022; 292: 114523. DOI: 10.1016/j.socscimed.2021.114523. 34785096

[23] Pearson A . Being honest. Nurs Stand 2006; 21: 22–23. 10.7748/ns.21.3.22.s26 17036733

[24] Myburgh H Calitz E Railton JP , et al. Breaking down barriers to tell: a mixed methods study of health worker involvement in disclosing to children that they are living with HIV in rural South Africa. J Assoc Nurses AIDS Care 2018; 29: 902–913. DOI: 10.1016/j.jana.2018.04.009. 29784521

[25] Liamputtong P. Qualitative research methods. In: Naomi S (Ed.). Docklands. 5th ed. VIC: Oxford University Press, 2020.

[26] Richardson-Tench M Taylor BJ Kermode S , et al. Research in nursing: evidence for best practice. 5th ed.: South Melbourne, VIC: Cengage Learning, 2014.

[27] Braun V Clarke V . Using thematic analysis in psychology. Qual Res Psychol 2006; 3: 77–101. DOI: 10.1191/1478088706qp063oa.

[28] Lieber E Weisner TS . Dedoose Version 9.0.107, cloud application for managing, analyzing, and presenting qualitative and mixed method research data. Los Angeles, CA: Sociocultural Research Consultants, LLC, 2023. www.dedoose.com.

[29] Smith JA Flowers P Larkin M . Interpretative phenomenological analysis : theory, method and research. 2nd ed. Great Britain: Sage Publications, 2022.

[30] Guba EG . Criteria for assessing the trustworthiness of naturalistic inquiries. Educ Commun Technol 1981; 29: 75. DOI: 10.1007/BF02766777.

[31] Lincoln YS Guba EG . Naturalistic inquiry. Beverly Hills, Calif: Sage Publications, 1985.

[32] El Ali M O’Neill J Gillam L . Paediatric Nurses’ Personal Accounts of Being Told Not to Disclose Information to Children With Serious Illness—An Interpretative Phenomenological Study. Journal of advanced nursing 2024. DOI: 10.1111/jan.16596. 39494747

[33] Nursing and Midwifery Board Ahpra . Registered nurse standards for practice. Australia: Nursing and Midwifery Board of Australia, 2017. https://www.nursingmidwiferyboard.gov.au/Codes-Guidelines-Statements/Professional-standards/registered-nurse-standards-for-practice.aspx#.

[34] Olson LL Stokes F. American Nurses Association. The ANA Code of Ethics for Nurses With Interpretive Statements: Resource for Nursing Regulation. Journal of nursing regulation 2016; 7: 9–20. DOI: 10.1016/S2155-8256(16)31073-0.

[35] Martakis K Brand H Schröder-Bäck P . Developing child autonomy in pediatric healthcare: towards an ethical model. Arch Argent Pediatr 2018. DOI: 10.5546/aap.2018.eng.e401. 29756714

[36] Archard D . Children: rights and childhood. 3rd ed. New York: Routledge, Taylor & Francis Group, 2015.

[37] British Medical Association . Consent, rights and choices in health care for children and young people. TavistockSquare, London WC1H 9JR: BMJ Books, BMA House, 2001.

[38] UNHR-OHC . Convention on the rights of the child . The United Nations. 1989. https://www.ohchr.org/en/instruments-mechanisms/instruments/convention-rights-child.

[39] Shahriari M Mohammadi E Abbaszadeh A , et al. Nursing ethical values and definitions: a literature review. Iran J Nurs Midwifery Res 2013; 18: 1–8. 23983720 PMC3748548

[40] Kopec M . Deceptive omissions, half-truths, and the moral exemplar in clinical ethics. Am J Bioeth 2021; 21: 33–35. DOI: 10.1080/15265161.2021.1906993. 33945420

[41] Driver J . Uneasy virtue. 1st ed. Cambridge, UK: Cambridge University Press, 2001.

[42] Curzer HJ . Aristotle and the virtues. Oxford: Oxford University Press, 2012.

[43] Hursthouse R Pettigrove G . Virtue ethics. In: Zalta EN (Ed.). Stanford Encyclopedia of Philosophy [Internet]. Stanford (CA): Metaphysics Research Lab, Stanford University; 2022. Available from: https://plato.stanford.edu/entries/ethics-virtue/.

[44] May C . Disclosure of terminal prognoses in a general hospital: the nurse’s view. J Adv Nurs 1993; 18: 1362–1368. DOI: 10.1046/j.1365-2648.1993.18091362.x. 8258593

[45] Ricciardelli R Johnston MS Bennett B , et al. “It is difficult to always Be an antagonist”: ethical, professional, and moral dilemmas as potentially psychologically traumatic events among nurses in Canada. Int J Environ Res Publ Health 2022; 19: 1454. DOI: 10.3390/ijerph19031454. PMC8834915 35162485

[46] Lupton D . Perspectives on power, communication and the medical encounter: implications for nursing theory and practice. Nurs Inq 1995; 2: 157–163. DOI: 10.1111/j.1440-1800.1995.tb00166.x. 7664160

